# Comparative analysis of module-based versus direct methods for reverse-engineering transcriptional regulatory networks

**DOI:** 10.1186/1752-0509-3-49

**Published:** 2009-05-07

**Authors:** Tom Michoel, Riet De Smet, Anagha Joshi, Yves Van de Peer, Kathleen Marchal

**Affiliations:** 1 Department of Plant Systems Biology, VIB, Technologiepark 927, B-9052 Gent, Belgium; 2 Department of Molecular Genetics, Ghent University, Technologiepark 927, B-9052 Gent, Belgium; 3 CMPG, Department Microbial and Molecular Systems, KU Leuven, Kasteelpark Arenberg 20, B-3001 Leuven, Belgium

## Abstract

**Background:**

A myriad of methods to reverse-engineer transcriptional regulatory networks have been developed in recent years. Direct methods directly reconstruct a network of pairwise regulatory interactions while module-based methods predict a set of regulators for modules of coexpressed genes treated as a single unit. To date, there has been no systematic comparison of the relative strengths and weaknesses of both types of methods.

**Results:**

We have compared a recently developed module-based algorithm, LeMoNe (Learning Module Networks), to a mutual information based direct algorithm, CLR (Context Likelihood of Relatedness), using benchmark expression data and databases of known transcriptional regulatory interactions for *Escherichia coli *and *Saccharomyces cerevisiae*. A global comparison using recall versus precision curves hides the topologically distinct nature of the inferred networks and is not informative about the specific subtasks for which each method is most suited. Analysis of the degree distributions and a regulator specific comparison show that CLR is 'regulator-centric', making true predictions for a higher number of regulators, while LeMoNe is 'target-centric', recovering a higher number of known targets for fewer regulators, with limited overlap in the predicted interactions between both methods. Detailed biological examples in *E. coli *and *S. cerevisiae *are used to illustrate these differences and to prove that each method is able to infer parts of the network where the other fails. Biological validation of the inferred networks cautions against over-interpreting recall and precision values computed using incomplete reference networks.

**Conclusion:**

Our results indicate that module-based and direct methods retrieve largely distinct parts of the underlying transcriptional regulatory networks. The choice of algorithm should therefore be based on the particular biological problem of interest and not on global metrics which cannot be transferred between organisms. The development of sound statistical methods for integrating the predictions of different reverse-engineering strategies emerges as an important challenge for future research.

## Background

Due to the success of microarray technology, the available data on the transcriptional regulatory networks of different organisms has grown exponentially. In order to explore these data to the maximum, a myriad of methods to reverse-engineer or reconstruct transcriptional regulatory networks from microarray data have been developed in the past few years. In general, the scientific community has mainly focused on the overall performance of newly developed methods in reconstructing the known network of certain model organisms as compared to a reference network, measuring algorithmic performance with standard measures such as recall and precision. Less attention has been paid to what extent conceptually different approaches differ in the networks they infer. Nonetheless, in order to get a better understanding of the systems studied it is also important to understand which specific problems can be tackled using a certain method, irrespective of the overall performance of the different methods.

Broadly speaking we can distinguish between two classes of methods for reverse-engineering transcriptional regulatory networks from gene expression data which differ vastly in how they approach the network inference problem. Direct methods infer individual regulator-target interactions using a pairwise correlation measure between the expression profiles of a transcription factor and its putative targets [1,2]. Module-based methods assume a modular structure of the transcriptional regulatory network [3-5], with genes subject to the same regulatory input being organized in coexpression modules.

While different direct methods have been compared to each other in the past [2,6,7], no systematic comparison between direct and module-based methods has been undertaken so far. In this study we perform such a comparison using a representative method from each class. The CLR (Context Likelihood of Relatedness) algorithm [2] considers all possible pairwise regulator-target interactions and scores these interactions based on the mutual information of their expression profiles as compared to an interaction specific background distribution. It has been shown to outperform other direct methods [2]. The LeMoNe (Learning Module Networks) algorithm [8] uses probabilistic, ensemble-based optimization techniques [8,9] to infer high-quality module networks [3], where genes are first partitioned into coexpression modules and regulators are assigned to modules based on how well they explain the condition-dependent expression behavior of the module. It has been shown to outperform the original module network algorithm [8]. We have compared both methods at increasing levels of detail using public expression compendia for *Escherichia coli *[2] and *Saccharomyces cerevisiae *[10], two organisms for which relatively large databases of known transcriptional regulatory interactions exist [11,12]. We first use recall versus precision curves to give a comparison of the global performance of both methods. We then show that due to the different assumptions underlying both methodologies, they infer topologically distinct networks with limited overlap, even at equal performance thresholds. To understand these distinctions more completely, we examined in detail example subsystems of the network which are well characterized, namely the chemotaxis and flagellar system in *E. coli *and a respiratory module and a membrane lipid and fatty acid metabolism module in *S. cerevisiae*. Biological validation of the inferred networks cautions against over-interpreting recall and precision values computed using incomplete reference networks.

## Results and discussion

### Global comparison using recall and precision

**Figure 1:**
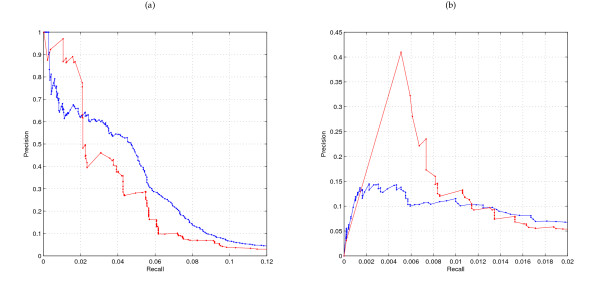
**Global comparison of LeMoNe and CLR using recall versus precision curves**. Recall versus precision curves for LeMoNe (red) and CLR (blue) for *E. coli *(a) and *S. cerevisiae *(b). Note the difference in scale between both organisms.

The output of LeMoNe and CLR consists of a list of respectively ranked regulator-module and ranked regulator-target interactions, scored according to their statistical significance. As a first, global comparison, we can therefore compute recall and precision with respect to the given reference networks at different score cutoffs. For CLR we can directly compare the inferred network with the true network; for LeMoNe we draw an edge between each regulator assigned to a module and all genes in the module, thereby ignoring at this stage the extra information present in the module structure. We computed recall and precision as in [2]: if an edge is predicted between two genes present but unconnected in the reference network it is counted as a false positive. Figure 1 shows the recall versus precision curves for both algorithms and both organisms. Both algorithms succesfully prioritize true positive interactions, especially in *E. coli*: all curves go from a high precision, low recall region to a low precision, high recall region. For CLR the curves show a smooth course while for LeMoNe they are more staircase-like. CLR scores individual interactions and as a result, in the recall-precision curve interactions will be added one by one, but interactions corresponding to a certain regulator will be dispersed continuously throughout the recall-precision curve. LeMoNe on the other hand assigns a regulator to a module as a whole and all targets belonging to the same module are added at the same time in the recall-precision curve. For a stringent threshold and subsequently a low number of interactions inferred, the CLR network will cover few interactions for many regulators while the LeMoNe network will retrieve many interactions for few regulators.

At similar levels of precision, the recall in *S. cerevisiae *is nearly an order of magnitude smaller than in *E. coli*, in line with previous studies [13]. This is likely due to the higher complexity of transcriptional regulation in *S. cerevisiae *with a higher degree of combinatorial regulation and posttranscriptional control, and consequently a lower degree of correlation in expression between transcription factors and their targets. A simple 'area under the curve' measurement would suggest that CLR performs slightly better in the prokaryote *E. coli *and LeMoNe in the eukaryote *S. cerevisiae*. However, as we will show below, both algorithms infer complementary information in both organisms.

### Topological distinctions between inferred networks

As explained in the previous section, due to how interactions are scored, direct and module-based methods will infer different kinds of networks at stringent precision thresholds. For *E. coli*, we compared the LeMoNe and CLR networks at a 30% precision threshold where both networks have nearly equal recall and precision (see Figure 1). The LeMoNe network consists of 53 regulators assigned to 62 modules for a total of 1079 predicted interactions; 594 of these interactions are between genes in RegulonDB, with a precision of 29%. The corresponding CLR network contains 1422 predicted interactions for 242 regulators; 597 of these interactions are between genes in RegulonDB, with a precision of 30%. 51 out of 53 LeMoNe regulators are also present in the CLR network, but only 277 interactions are predicted in both networks. For *S. cerevisiae*, there is no 'natural' point on the recall versus precision curve to compare both networks. We therefore compared CLR and LeMoNe at the first 1070 predicted interactions. This number is chosen to give comparably sized networks as in *E. coli *and ensure that the ranked list of LeMoNe interactions is not cut off in the middle of one module. The cutoff of the first 1070 interactions corresponds to precision values of respectively 16% and 10% for LeMoNe and CLR (cfr. Figure 1). The LeMoNe network consists of 34 regulators assigned to 39 modules containing 867 genes, while the CLR network contains 214 regulators; 28 regulators are present in both networks, yet only 75 interactions are common.

**Figure 2:**
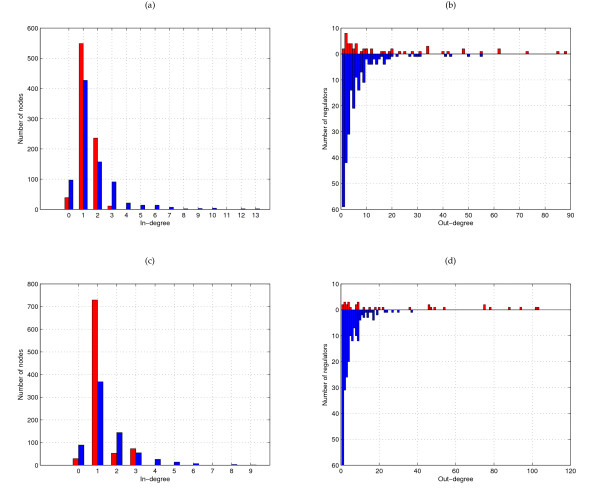
**In- and out-degree distributions of LeMoNe and CLR networks**. **(a) ***E. coli *in-degree distribution for LeMoNe (red) and CLR (blue) at 30% precision threshold. **(b) ***E. coli *out-degree distribution for LeMoNe (red) and CLR (blue) at 30% precision threshold. **(c) ***S. cerevisiae *in-degree distribution for LeMoNe (red) and CLR (blue) at first 1070 predictions. **(d) ***S. cerevisiae *out-degree distribution for LeMoNe (red) and CLR (blue) at first 1070 predictions.

The networks inferred by LeMoNe and CLR are topologically very distinct (see Additional File 1, Additional File 2, Additional File 3 and Additional File 4). This distinction can be quantified by their in-and out-degree distributions (Figure 2). The in-degree is the number of regulators assigned to a certain target gene and the in-degree distribution counts for each value *k *the number of targets with in-degree *k*. Likewise, the out-degree is the number of targets assigned to a certain regulator and the out-degree distribution counts for each value *k *the number of regulators with out-degree *k*. CLR infers for each regulator only the most significant targets. As a result, the out-degree distribution is skewed to the left, with the majority of regulators having only few targets. The in-degree distribution on the other hand has a long tail of genes assigned to many different regulators. LeMoNe infers for each module the most significant regulators, resulting in opposite characteristics of the degree distributions. The in-degree distribution has no tail since for most modules at most 2 significant regulators are identified. The out-degree distribution on the other hand has a long tail since each regulator assignment involves a whole module of genes. For these reasons, we say that CLR is 'regulator-centric' and LeMoNe is 'target-centric'.

### Regulator specific comparison

We make a further comparison of the two methods, focusing on how they differ in the type of regulators they assign. We compared again the 30% precision networks for *E. coli *and the networks of first 1070 interactions for *S. cerevisiae*.

For both methods, a large fraction of the regulators for which known targets are inferred are autoregulators. For *E. coli*, LeMoNe and CLR have respectively 19 and 32 regulators with at least one true positive; 15/19 (79%) and 27/32 (84%) are known autoregulators, while the fraction of autoregulators in the total reference network is 95/150 (63%). For *S. cerevisiae*, LeMoNe and CLR have respectively 6 and 10 regulators with at least one true positive; 5/6 (83%) and 5/10 (50%) are known autoregulators, while the fraction of autoregulators in the total reference network is 79/171 (46%). The abundance of autoregulators is not surprising since autoregulation is a simple mechanism by which the expression profile of a regulator and its targets can be correlated.

In LeMoNe, we get as additional information whether a predicted regulator is positively or negatively correlated with its target module and RegulonDB, the reference network for *E. coli*, contains the activation or repression sign for many interactions. However, although theoretically possible, we could not detect biologically relevant patterns of anticorrelation, in line with previous studies [14]. Even though the assumption of anticorrelation seems intuitively plausible in case of repressors, it is a too simplistic representation of reality. Indeed LeMoNe and CLR both find many targets of mainly autorepressors (e.g. LexA, PurR, LldR and GalS), but they all were positively instead of negatively correlated with their targets. This can be explained by the fact that the activity of such autorepressors is dependent upon the presence of corepressing signals. In the absence of the corepressing signal the repressor is active, limiting its own production as well as that of its target genes. In presence of the corepressing signal the repressors are inactive, which enables the production of both inactive repressor gene and its targets [15-17].

**Figure 3:**
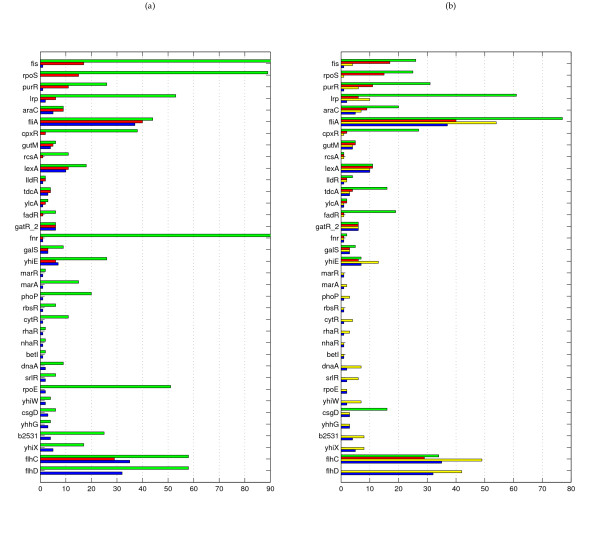
**Regulator specific comparison of LeMoNe and CLR on E. coli**. For each regulator in *E. coli *with known interactions inferred: **(a) **the number of interactions in the reference network (green) and the number of true positives in LeMoNe (red) and CLR (blue); **(b) **the number of interactions inferred (green) and the number of true positives (red) in LeMoNe, and the number of interactions inferred (yellow) and the number of true positives (blue) in CLR. LeMoNe and CLR networks are both at 30% precision threshold. Regulators are sorted by the difference TP_LeMoNe _– TP_CLR_. The total number of true positives is 171 for LeMoNe and 180 for CLR. For clarity, the *x*-axis in (a) is truncated, the true number of targets for Fis and Fnr is respectively 111 and 173. The number of interactions inferred only counts targets that belong to the reference network.

**Figure 4:**
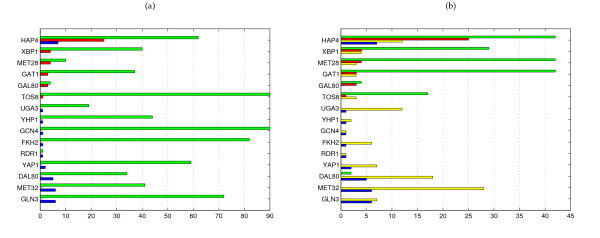
**Regulator specific comparison of LeMoNe and CLR on S. cerevisiae**. For each regulator in *S. cerevisiae *with known interactions inferred: **(a) **the number of interactions in the reference network (green) and the number of true positives in LeMoNe (red) and CLR (blue); **(b) **the number of interactions inferred (green) and the number of true positives (red) in LeMoNe, and the number of interactions inferred (yellow) and the number of true positives (blue) in CLR. LeMoNe and CLR networks are both cut off at the first 1070 predictions. Regulators are sorted by the difference TP_LeMoNe _– TP_CLR_. The total number of true positives is 40 for LeMoNe and 31 for CLR. For clarity, the *x*-axis in (a) is truncated, the true number of targets for GCN4 is 120. The number of interactions inferred only counts targets that belong to the reference network.

In *E. coli*, regulators for which the module-based and direct methods differ in performance are in line with the topological distinctions. CLR is better at inferring interactions for regulators that are known to regulate just one or a few operons (e.g. BetI, CsgD, DnaA, MarA, Yhhg, see Figure 3). These operons are found with a relatively high rank in the CLR network since their regulators often belong themselves to the operons and are thus by definition tightly coexpressed with their targets. The clustering method employed by LeMoNe appears to be too coarse grained to identify these operons individually, since they are mostly part of larger clusters. LeMoNe on the other hand is superior at inferring interactions for regulators that are known to regulate larger regulons, such as Fis, LexA, PurR, and RpoS, for which the level of coexpression is not as high as the one observed within a single operon (see Figure 3). In *S. cerevisiae*, there is no operonic structure and hence the 'operon regulators' acurately identified by CLR are absent. Figure 4 show however that the regulators for which LeMoNe and CLR infer known targets are still very distinct, but there appears to be no general biological reason underlying these differences.

### Biological validation of inferred networks

Due to the lack of a negative gold standard, we have denoted in the previous analysis an edge as being false positive if both regulator and target are present but not connected in the reference network (the positive gold standard). Since the coverage of these reference networks is still very incomplete, it is likely that the number of false positives is overestimated. Moreover, about half of the regulators in *E. coli *and *S. cerevisiae *are not present in the reference network and their predicted interactions are thus never evaluated. In [2], it was already shown that new predictions made by CLR in *E. coli *could be validated experimentally. Here we have performed an in-depth biological validation of the 30% precision module network inferred by LeMoNe. To biologically validate the obtained regulator-module assignments, we calculated for all modules functional enrichment scores [18] and enrichment in targets of previously annotated regulators [11]. Table 1 shows that in nearly all cases the module is enriched in known targets of the predicted regulator (column 4) or at least involved in the same biological function (column 6). In several cases the predicted regulator is the one which has the best target enrichment *p*-value. Nearly half of the regulators are putative regulators without any currently known targets, and these assignments cannot be validated. However, many of the correctly predicted regulators involve neighbor regulators [19] (Table 1, column 7), i.e. regulators colocalized with their targets on the genome. It has been suggested that many of the putative regulators in *E. coli *constitute such neighbor regulators [20]. Hence this feature of gene neighborhood can be used to attach additional significance to the high-scoring predictions for uncharacterized regulators. One of the advantages of a module-based approach is the fact that if a certain module contains several known targets of the assigned regulators, the rest of the unknown targets in this module can be considered high confidence predictions for that regulator. This is illustrated in Additional File 5, where we list several predictions for 10 different modules which could be confirmed by a thorough literature search.

**Table 1:** Biological validation of LeMoNe on E. coli

Regulator	Module ID	Score	Target enrich.	Autoreg.	Pathway	Local	Function
gatR_2	73	1912.98	**		*	**	carbon utilization > carbon compounds
gadE	48	1844.50	**	*	*	**	adaptations > pH
gutM	38	1807.24	**	*	*	*	carbon utilization > carbon compounds
**ymfN**	**58**	1749.11				*	
**ymfN**	**33**	1711.17				*	
fliA	12	1510.48	**	*	*	**	motility, chemotaxis, energytaxis; flagella; biosynthesis of flagellum
rcsB	**62**	1261.72			*	*	biosynthesis of colanic acid (M antigen)
fecI	57	1200.77		*	*		adaptations > Fe aquisition
gatR_2	42	1176.55	**		*	**	carbon utilization > carbon compounds
**yahA**	82	1171.92					
rcsA	87	1151.97	**	*	*		biosynthesis of colanic acid (M antigen)
lexA	20	996.62	**	*	*	*	SOS response; DNA repair; protection > radiation
lldR	65	976.84	**	*	*	*	energy metabolism; aerobic respiration
fliA	45	956.70	**	*	*		motility, chemotaxis, energytaxis
fliA	18	903.46	*	*	*		biosynthesis of flagellum; motility, chemotaxis, energytaxis; flagella
nac	85	827.17		*	*		nitrogen metabolism
**yiaG**	15	816.55					
**ydaK**	23	815.75				**	
**ydaK**	154	805.22					
fnr	23	798.27	*	*	*	**	energy metabolism; anaerobic respiration; membrane
lrp	5	777.80		*	*		biosynthesis of building blocks > amino acids
araC	46	760.44	**	*	*	**	carbon utilization > carbon compounds
appY	50	748.75					
**yfiE**	**67**	736.50					
**osmE**	15	734.87					
lexA	78	726.67	**	*	*		SOS response
purR	144	708.63		*	*		
uidR	81	708.36		*			
araC	21	678.10	*	*	*		carbon utilization > carbon compounds
**yfeG**	29	663.94					
**b1450**	**53**	662.16					
flhC	18	650.64	**		*		biosynthesis of flagellum; motility, chemotaxis, energytaxis; flagella
**ogrK**	83	645.35					
fliA	17	637.28		*			
rpoS	14	637.13	**		*	*	adaptations > osmotic pressure
pdhR	**55**	633.52		*	*		energy metabolism; anaerobic respiration
tdcA	31	619.06	*	*	*	*	threonine catabolism; carbon utilization > amino acids
**yebK**	**106**	617.44					
araC	56	608.17	**	*	*		carbon utilization > carbon compounds
csgD	26	599.30		*			
hycA	66	596.27					
tdcR	11	593.75			*		carbon utilization > amino acids
fliA	24	593.05	*	*	*		flagella; motility, chemotaxis, energytaxis; biosynthesis of flagellum
chbR	24	590.31		*			
hycA	29	563.45				*	
galS	76	561.25	**	*	*	**	carbon utilization > carbon compounds
**nlp**	77	559.41					
**yfeC**	119	549.33					
**b1506**	36	548.33					
lrp	10	528.90	*	*	*		biosynthesis of building blocks > amino acids
**cspB**	37	527.86					
cusR	68	515.56	**	*	*	**	extrachromosomal > transposon related
**b1284**	51	514.78					
nanR	9	508.87					
**yohL**	90	496.21					
lrp	126	493.60		*	*		biosynthesis of building blocks > amino acids
**yjjQ**	**179**	491.02					
**yehV**	**63**	483.29					
**ogrK**	27	481.75					
slyA	3	474.43					
**ydcN**	16	467.66					
cpxR	9	465.39	*	*	*		adaptations > other (mechanical, nutritional, oxidative stress)
**yehV**	34	451.77					
fruR	**63**	449.25					
araC	64	441.57	*	*	*		carbon utilization > carbon compounds
fis	19	436.12	**	*	*	*	information transfer > RNA related > tRNA
fadR	16	435.98	*				
purR	10	431.78	**	*	*		biosynthesis of building blocks > nucleotides
cadC	37	429.32		*			
fecI	54	429.28		*			
**rstA**	**102**	428.94					
tdcR	**61**	428.84					
flhC	24	426.88	**		*	*	flagella; motility, chemotaxis, energytaxis; biosynthesis of flagellum

Biological validation of the LeMoNe 30% precision network for *E. coli*. Target enrichment: (*) module is enriched in known targets of the predicted regulator, (**) module is most enriched for predicted regulator. Autoregulator: (*) regulator is an autoregulator. Pathway: (*) module is enriched in the same function(s) as the regulator. Local: (*) regulator is in the same operon as the module genes, (**) Transcription unit of regulator is adjacent to transcription units of the module genes. Function: enriched functions of the module. Regulators in bold face are putative regulators without known targets; module IDs in bold face consist only of uncharacterized genes.

Module network predictions in *S. cerevisiae *have been experimentally validated in [3] and functionally analysed in [3,8]. For further validation we compared the CLR and LeMoNe networks to the YEASTRACT database [21]. This database contains most of the interactions in the reference network we use here [12]. In addition it also contains targets inferred by transcription factor deletion microarray experiments. The number of true positives for the LeMoNe network cut off at the first 1070 predictions increases from 40 (precision 16%) in the reference network to 55 (precision 24%) with respect to YEASTRACT. For the CLR network cut off at the first 1070 predictions, the number of true positives increases from 31 (precision 10%) in the reference network to 48 (precision 12%) with respect to YEASTRACT.

Biological validation of inferred networks is tedious and does not provide an easy alternative to the automatic estimation of true and false positives using an established reference network. The results of this section do show however that many 'false positives' with respect to an incomplete network are actually true positives when additional information is taken into account and that recall versus precision plots such as in Figure 1 have to be interpreted with caution.

### The chemotaxis and flagellar system in Escherichia coli

**Figure 5:**
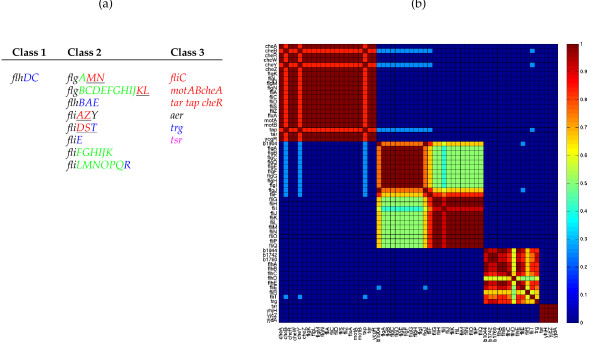
**Condition specific clustering in the chemotaxis and flagellar system in E. coli**. **(a) **Operons encoding the proteins of the chemotaxis and flagellar system in *E. coli*. The underlined genes belong to operons activated by FlhDC but have additional promoters activated by FliA. They are expressed partially as class 2 genes and fully as class 3 genes. Table and data after [22]. Genes belonging to module 12 are indicated in red, to module 18 in green, to module 24 in blue and to module 45 in magenta. **(b) **Pairwise clustering frequencies in the LeMoNe clustering ensemble [8,9] for the flagella genes. Each row/column corresponds to a gene in one of the flagella modules and the heat map value at position (*i, j*) is the frequency with which gene *i *and *j *cluster together. The blocks along the diagonal correspond to respectively module 12, 18, 24 and 45. In module 24, it can be seen that the coclustering frequencies of *flhD *with the other members is rather low, indicating a weaker degree of coexpression. See also Supplementary Figure S5.

Our analysis has shown that at equal levels of recall and precision, LeMoNe predicts interactions for fewer regulators but with higher coverage per regulator while CLR predicts fewer interactions per regulator but for more regulators. It is instructive to analyse in detail the implications of these differences for subsystems of the transcriptional regulatory network which are particularly well perturbed in the data set. For *E. coli*, we have taken a closer look at the chemotaxis and flagellar system which forms a complex and tightly regulated system. It consists of the class 1 master operon *flhDC*, 8 class 2 operons activated by the complex FlhDC, and at least 6 class 3 operons activated by the sigma factor FliA (Figure 5(a)). The *fliA *operon belongs to class 2, positively regulates its own production and can activate other class 2 operons as well [22].

Four modules (12, 18, 24 and 45) in the module network are enriched in flagellar functions. Together they contain 60 genes of which 55 are known flagellar genes. The separation of flagellar genes in different modules is strongly supported by the LeMoNe clustering (Figure 5(b)), suggesting the presence of condition-specific regulation in the flagellar gene network, and corresponds to the difference in regulatory input between different classes of flagellar genes (Figure 5, see also Additional File 6). In the 30% precision LeMoNe network, FliA is assigned to all four modules and FlhC is correctly assigned to the class 2 modules 18 and 24 only. FlhD is not assigned with a score high enough to make the threshold.

At the 30% precision cutoff, LeMoNe and CLR agree for the majority of predicted interactions for FliA and FlhC. In addition, CLR infers several correct targets for FlhD. The coexpression of FlhD with its predicted targets is significantly lower than for FliA or FlhC. This is evidenced for instance from the LeMoNe clustering (Figure 5(b)) or CLR mutual information values (data not shown). However, due to the regulator-centric viewpoint and the 'local' background correction method of CLR, these relatively weakly coexpressed targets still get a significant mutual information *z*-score and are thus part of the predicted network. In the target-centric LeMoNe network, the potential assignment of FlhD to the flagella modules is compared to the much better scoring assignments of FliA and/or FlhC and therefore not deemed significant enough. Hence the regulator-centric CLR approach has the advantage to identify significant targets for all three flagellar regulators, but does not distinguish well between regulation by FlhDC and FliA due to the large overlap in predicted targets. The target-centric LeMoNe approach on the other hand has the advantage to infer detailed condition-specific regulatory information through the division in distinct modules of the flagellar genes, but only infers targets for FliA and FlhC.

### The respiratory module and membrane lipid and fatty acid metabolism module in Saccharomyces cerevisiae

Despite the overall low performance on *S. cerevisiae*, LeMoNe and CLR both achieve good results on particular subsystems. The advantage of a target-centric approach is well exhibited by the respiratory system. This system is well perturbed in the data set and clusters of respiratory genes are found repeatedly in it using various approaches [3,8,23]. LeMoNe module 7 contains 30 genes of which 23 are known respiratory genes. Hap4, a global regulator of respiratory genes, is the most significant regulator for this module and indeed 25 of its genes are known Hap4 targets. The pairwise correlation between Hap4 and its targets varies, and since CLR scores all interactions individually, they are dispersed throughout the ranked list of interactions. As a result, there are only 12 predicted Hap4 targets (7 TP) in the first 1070 CLR interactions (see also Figure 4). Clearly, the preliminary step of clustering genes into target modules was necessary here to infer the complete Hap4 regulated module.

**Figure 6:**
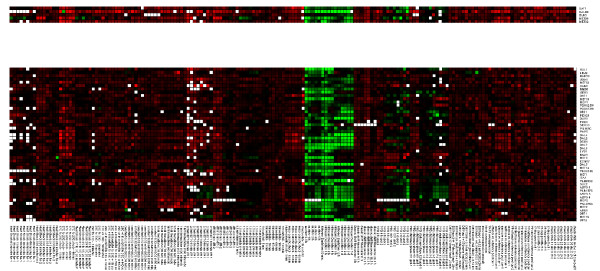
**Coupling of the methionine pathway and the nitrogen catabolite repression system in S. cerevisiae predicted by integration of LeMoNe and CLR networks**. LeMoNe module 11 with genes (bottom) and predicted regulators (top) involved in the methionine pathway (regulated by Met28 and Met32) and the nitrogen catabolite repression system (regulated by Gat1, Dal80 and Gln3). The regulators are, from top to bottom: Gat1, predicted by LeMoNe, 1 target predicted by CLR; Dal80, 11 targets predicted by CLR; Gln3, 6 targets predicted by CLR; Met28, predicted by LeMoNe, 4 targets predicted by CLR; Met32, 23 targets predicted by CLR. The upregulated (green) conditions are all amino acid starvation or nitrogen depletion conditions.

Another interesting example is given by LeMoNe module 11, a module of 47 genes involved in membrane lipid and fatty acid metabolism. The four highest-ranked regulators by LeMoNe for this module (Gat1, Met28, Met32 and Dal80) all have known targets in it. However, due to how regulators are scored in LeMoNe, there are rarely more than two significant regulators per module (see Figure 2(a) and 2(c)), and only the assignments of Gat1 (3 TP) and Met28 (4 TP) are present in the network of the first 1070 LeMoNe interactions. CLR on the other hand finds the most significant targets for each regulator individually and thus identifies correct targets from module 11 for the other regulators as well: Met28 (1 TP), Met32 (6 TP) and Dal80 (6 TP). For Gat1, CLR does not find true positives, however it finds 5 TP in module 11 for a fifth regulator Gln3. Hence for this module, the most complete information is retrieved by combining the output of LeMoNe and CLR. The genes and predicted regulators of module 11 are mostly involved in 2 pathways, the methionine pathway (regulated by Met28 and Met32) and the nitrogen catabolite repression (NCR) system (regulated by Gat1, Dal80 and Gln3). Module 11 is overexpressed in nitrogen depletion and amino acid starvation conditions (see Figure 6). For NCR-sensitive genes it is known that they are not activated when rich nitrogen sources are available, but get expressed when only poor sources are left. A link between the methionine pathway and nitrogen depletion, as predicted by LeMoNe through the clustering and by CLR through the assignment of common targets to these regulators, is not evident but appears to be confirmed by an ongoing study [24].

## Conclusion

In recent years, a wide variety of methods to reverse-engineer transcriptional regulatory networks from microarray data have been developed. Whereas the development of a new method mostly coincides with a comparison in overall performance to all existing methods, so far no in-depth study on how conceptual differences relate to differences in the inferred networks have been made. Here we distinguished between two main approaches for reverse-engineering transcriptional regulatory networks: the module-based approach and the direct approach. We compared a representative algorithm of each approach (module based LeMoNe versus direct CLR) at several levels of detail for two different organisms, the prokaryote *E. coli *and the eukaryote *S. cerevisiae*. We have found that CLR is 'regulator-centric', making few but highly significant predictions for a large number of regulators. LeMoNe on the other hand is 'target-centric', identifying few but highly significant regulators for a large number of genes grouped in coexpression modules. Through a regulator specific comparison and analysis of specific biological subsystems, we have shown that at stringent significance cutoffs, the conceptual differences in statistically scoring potential regulatory interactions lead to topologically distinct inferred networks containing different kinds of regulators and biological information. Our results show that the choice of algorithm should be made primarily based on whether the biological question under study falls within the target-centric or regulator-centric viewpoint, and not on global metrics which cannot be transferred between organisms. Ideally, several network inference strategies should be combined for the best overall performance. It is an important challenge for future research to develop sound statistical methods for optimally combining the output of multiple, existing reverse-engineering algorithms.

## Methods

The *E. coli *microarray data compendium [2] contains expression profiles for 4345 genes under 189 different stress conditions and genetic perturbations. We selected a subset of 1882 differentially expressed genes (standard deviation larger than 0.5) and used a list of 316 known or putative transcription factors [11,18] to reconstruct regulatory networks. LeMoNe [8] (software available at ) identified 108 ensemble-averaged modules from 12 independent Gibbs sampler runs, containing 1761 genes in total. It inferred a ranked list of regulator-module edges from an ensemble of 10 regulatory programs per module with 100 regulator samples per regulatory program node (see [8] for more details on the meaning of these parameters). We applied CLR [2] (software available at ) on the data for the 2084 selected genes (the union of the 1882 differentially expressed genes and 316 candidate regulators) and kept all mutual information *z*-scores between the 316 transcription factors and 1882 target genes. As a reference network we used RegulonDB version 5.7 [11], a database of 4840 known transcriptional interactions in *E. coli *between 167 transcription factors and 1693 genes. Recall values are computed with respect to RegulonDB restricted to the subset of 2084 genes. This subnetwork contains 3110 edges between 150 transcription factors and 1053 genes. We used EcoCyc [18] to compute functional enrichment of modules. Target and functional enrichment in Table 1 were computed using a cumulative hypergeometric distribution, Bonferroni corrected for multiple testing, with confidence level 95%.

The *S. cerevisiae *microarray data compendium [10] contains expression profiles for 6153 genes in 173 different stress conditions. We used the same subset of 2355 differentially expressed genes, including a list of 321 potential regulators, as used in previous studies of this data set [3,8]. LeMoNe was run with the same settings as for *E. coli *and inferred 55 ensemble-averaged modules containing 1075 genes. As reference network we used a network recently compiled from the results of genetic, biochemical and ChIP-chip experiments [12]. It contains 11785 interactions between 154 transcription factors and 4047 genes. After restriction to the subset of 2355 differentially expressed genes, it contains 4513 interactions between 133 transcription factors and 1628 genes. The YEASTRACT [21] database contains 30979 transcriptional interactions in *S. cerevisiae *between 171 transcription factors and 5727 genes. After restriction to the subset of 2355 differentially expressed genes, it contains 12021 interactions between 137 transcription factors and 2182 genes (Additional files 7, 8, 9, 10, 11, 12, 13, 14, 15 and 16).

## Authors' contributions

TM developed software, analyzed data and wrote the manuscript. RDS analyzed data and wrote the manuscript. AJ developed software and analyzed data. YVdP supervised the study. KM wrote the manuscript and supervised the study. All authors read and approved the final manuscript.

## Supplementary Material

Additional file 1 **LeMoNe network for *E. coli *at 30% precision cutoff**. Supplementary Figure S1. Click here for file

Additional file 2 **CLR network for *E. coli *at 30% precision cutoff**. Supplementary Figure S2. Click here for file

Additional file 3 **LeMoNe network for *S. cerevisiae *at first 1070 predictions**. Supplementary Figure S3. Click here for file

Additional file 4 **CLR network for *S. cerevisiae *at first 1070 predictions**. Supplementary Figure S4. Click here for file

Additional file 5 **New interactions predicted in the 30% precision LeMoNe network validated by literature search**. Supplementary Table S1. Click here for file

Additional file 6 **Expression levels of chemotaxis and flagellar genes in *E. coli***. Supplementary Figure S5. Click here for file

Additional file 7 ***E. coli *list of candidate regulators**. *E. coli *list of candidate regulators, used as input for LeMoNe and CLR. Click here for file

Additional file 8 ***E. coli *reference network**. *E. coli *reference network of known transcriptional interactions, compiled from RegulonDB and EcoCyc. Click here for file

Additional file 9 ***E. coli *modules in the LeMoNe 30% precision network**. Gene content of *E. coli *LeMoNe modules in the 30% precision network. Click here for file

Additional file 10 ***E. coli *regulator to module assignments in the LeMoNe 30% precision network**. List of *E. coli *regulator to module assignments and their score in the LeMoNe 30% precision network. Click here for file

Additional file 11 ***E. coli *CLR 30% precision network**. List of *E. coli *interactions and their score in the CLR 30% precision network. Click here for file

Additional file 12 ***S. cerevisiae *list of candidate regulators**. *S. cerevisiae *list of candidate regulators, used as input for LeMoNe and CLR. Click here for file

Additional file 13 ***S. cerevisiae *reference network**. *S. cerevisiae *reference network of known transcriptional interactions, obtained from reference [12]. Click here for file

Additional file 14 ***S. cerevisiae *modules in the LeMoNe 1070 network**. Gene content of *S. cerevisiae *LeMoNe modules in the 1070 network. Click here for file

Additional file 15 ***S. cerevisiae *regulator to module assignments in the LeMoNe 1070 network**. List of *S. cerevisiae *regulator to module assignments and their score in the LeMoNe 1070 network. Click here for file

Additional file 16 ***S. cerevisiae *CLR 1070 network**. List of *S. cerevisiae *interactions and their score in the CLR 1070 network. Click here for file
