# Supplementary material for: Comparative analysis of module-based versus direct methods for reverse-engineering transcriptional regulatory networks
Source: BMC Syst Biol. 2009 May 7;3:49. doi: 10.1186/1752-0509-3-49 (PMC2684101; doi:10.1186/1752-0509-3-49)
Supplement: Additional file 2 — CLR network for E. coli at 30% precision cutoff. Supplementary Figure S2. [file 1752-0509-3-49-S2.pdf]

Supplementary Figure S2 – CLR network for *E. coli* at 30% precision cutoff

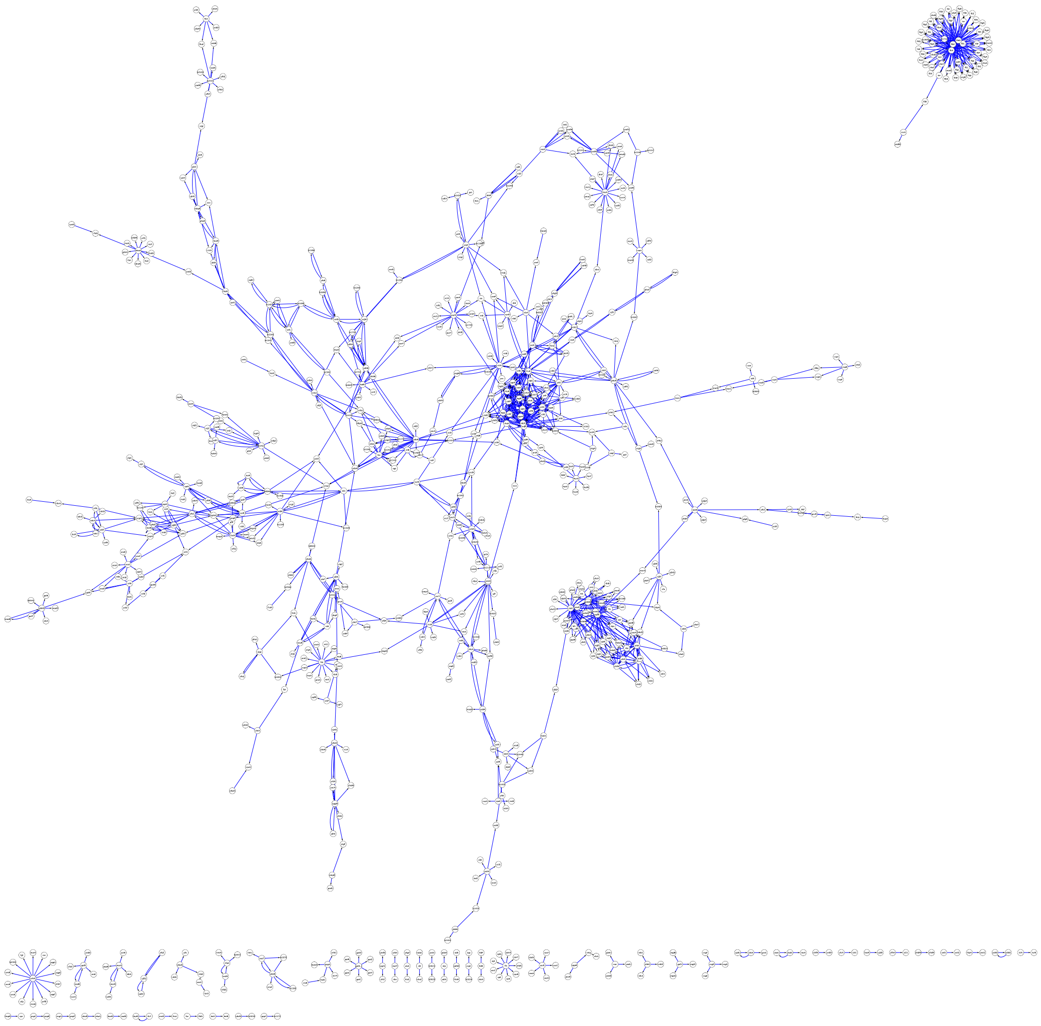

Figure S2: CLR network for *E. coli* at 30% precision cutoff.
