# Supplementary material for: Comparative analysis of module-based versus direct methods for reverse-engineering transcriptional regulatory networks
Source: BMC Syst Biol. 2009 May 7;3:49. doi: 10.1186/1752-0509-3-49 (PMC2684101; doi:10.1186/1752-0509-3-49)
Supplement: Additional file 4 — CLR network for S. cerevisiae at first 1070 predictions. Supplementary Figure S4. [file 1752-0509-3-49-S4.pdf]

Supplementary Figure S4 – CLR network for *S. cerevisiae* at first 1070 predictions

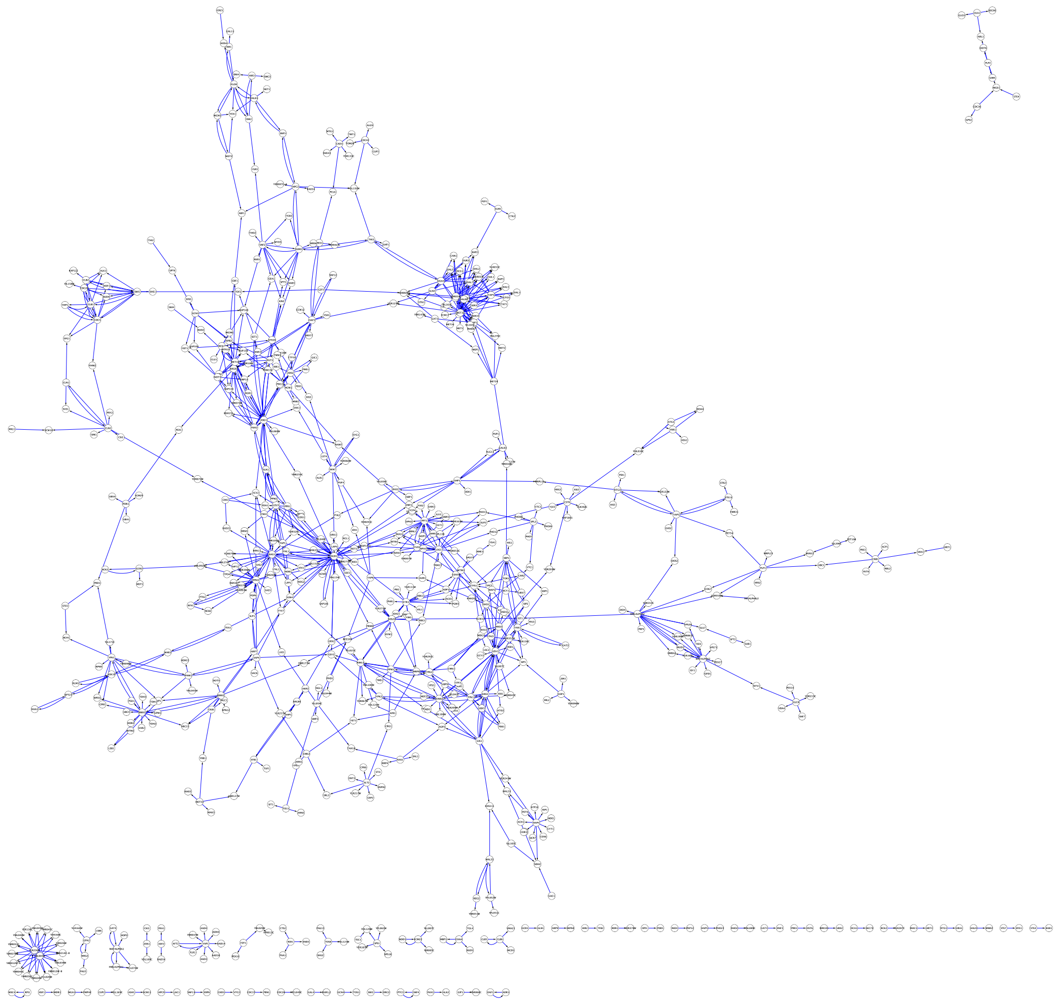

Figure S4: CLR network for *S. cerevisiae* at first 1070 predictions.
