# Supplementary material for: Comparative analysis of module-based versus direct methods for reverse-engineering transcriptional regulatory networks
Source: BMC Syst Biol. 2009 May 7;3:49. doi: 10.1186/1752-0509-3-49 (PMC2684101; doi:10.1186/1752-0509-3-49)
Supplement: Additional file 5 — New interactions predicted in the 30% precision LeMoNe network validated by literature search. Supplementary Table S1. [file 1752-0509-3-49-S5.pdf]

# Supplementary Table S1 – New interactions predicted in the 30% precision LeMoNe network validated by literature search

| MODULE | REGULATOR | GENE | EVIDENCE TYPE | REFERENCE |
|---|---|---|---|---|
| 5 | Lrp | gdhA | ChIP-qPCR | Faith et al. (2007) |
| 5 | Lrp | hisA | microarray analysis | Hung et al. (2002) |
| 5 | Lrp | hisB | microarray analysis | Hung et al. (2002) |
| 5 | Lrp | hisC | microarray analysis | Hung et al. (2002) |
| 5 | Lrp | hisD | microarray analysis | Hung et al. (2002) |
| 5 | Lrp | hisF | microarray analysis | Hung et al. (2002) |
| 5 | Lrp | hisG | microarray analysis | Hung et al. (2002) |
| 5 | Lrp | hisH | microarray analysis | Hung et al. (2002) |
| 5 | Lrp | hisI | microarray analysis | Hung et al. (2002) |
| 5 | Lrp | pntA | ChIP-qPCR | Faith et al. (2007) |
| 5 | Lrp | pntB | ChIP-qPCR | Faith et al. (2007) |
| 5 | Lrp | ydiJ | microarray analysis | Hung et al. (2002) |
| 10 | Lrp | aroG | ChIP-qPCR | Faith et al. (2007) |
| 10 | Lrp | leuB | microarray analysis (indirect influence of Lrp on expression of LeuLABCD according to Landgraf et al. (1999)) | Hung et al. (2002) |
| 10 | Lrp | pheA | microarray analysis | Hung et al. (2002) |
| 10 | Lrp | pheL | microarray analysis | Hung et al. (2002) |
| 10 | Lrp | purM | ChIP-qPCR | Faith et al. (2007) |
| 10 | Lrp | thrA | ChIP-qPCR | Faith et al. (2007) |
| 10 | Lrp | thrB | ChIP-qPCR | Faith et al. (2007) |
| 10 | Lrp | thrC | ChIP-qPCR | Faith et al. (2007) |
| 10 | Lrp | yagU | ChIP-qPCR | Faith et al. (2007) |
| 14 | RpoS | amyA | microarray analysis | Weber et al. (2005) |
| 14 | RpoS | b0753 | microarray analysis | Weber et al. (2005) |
| 14 | RpoS | b1953 | microarray analysis | Weber et al. (2005) |
| 14 | RpoS | b2080 | microarray analysis | Weber et al. (2005) |
| 14 | RpoS | b2086 | microarray analysis | Weber et al. (2005) |
| 14 | RpoS | psiF | microarray analysis | Weber et al. (2005) |
| 14 | RpoS | wrbA | microarray analysis | Weber et al. (2005) |
| 14 | RpoS | ybaT | microarray analysis | Weber et al. (2005) |
| 14 | RpoS | ybaS | microarray analysis | Weber et al. (2005) |
| 14 | RpoS | ybaY | microarray analysis | Weber et al. (2005) |
| 14 | RpoS | ycaC | microarray analysis | Weber et al. (2005) |
| 14 | RpoS | yccJ | microarray analysis | Weber et al. (2005) |
| 14 | RpoS | ycgB | microarray analysis | Weber et al. (2005) |
| 14 | RpoS | ycgZ | microarray analysis | Weber et al. (2005) |
| 14 | RpoS | yeaG | microarray analysis | Weber et al. (2005) |
| 14 | RpoS | yedU | microarray analysis | Weber et al. (2005) |
| 14 | RpoS | ygjG | microarray analysis | Weber et al. (2005) |
| 14 | RpoS | yhbO | microarray analysis | Weber et al. (2005) |
| 14 | RpoS | ymgA | microarray analysis | Weber et al. (2005) |
| 14 | RpoS | ymgB | microarray analysis | Weber et al. (2005) |
| 14 | RpoS | yphA | microarray analysis | Weber et al. (2005) |
| 19 | Fis | argQ | microarray analysis | Bradley et al. (2007) |
| 19 | Fis | argV | microarray analysis | Bradley et al. (2007) |
| 19 | Fis | argZ | microarray analysis | Bradley et al. (2007) |
| 19 | Fis | dniR | microarray analysis | Bradley et al. (2007) |
| 19 | Fis | glyW | microarray analysis | Bradley et al. (2007) |
| 19 | Fis | secG | microarray analysis | Bradley et al. (2007) |
| 20 | LexA | dinD | microarray analysis, ChIP-chip analysis | Courcelle et al. (2002), Wade et al. (2005) |
| 20 | LexA | dinI | microarray analysis, ChIP-chip analysis | Courcelle et al. (2002), Wade et al. (2005) |
| 20 | LexA | yafN | microarray analysis (co-upregulation of neighbouring ORFS, along with dinB) | Courcelle et al. (2002) |
| 20 | LexA | yafO | microarray analysis (co-upregulation of neighbouring ORFS, along with dinB) | Courcelle et al. (2002) |
| 20 | LexA | yebF | microarray analysis (co-upregulation of neighbouring ORFS, along with yebG) | Courcelle et al. (2002) |
| 20 | LexA | yebG | in EcoCyc | Karp et al. (2007) |
| 20 | LexA | yigN | functional LexA-binding site (electrophoretic gel mobility shift assay), microarray analysis, ChIP-chip analysis | Fernández de Henestrosa et al. (2000), Courcelle et al. (2002), Wade et al. (2005) |
| 20 | LexA | yjiW | ChIP-chip analysis | Wade et al. (2005) |
| 23 | FNR | b1341 | ChIP-chip analysis | Grainger et al. (2007) |
| 23 | FNR | b1342 | ChIP-chip analysis | Grainger et al. (2007) |
| 23 | FNR | ydaA | ChIP-chip analysis | Grainger et al. (2007) |
| 24 | FliA | b1742 | microarray analysis | Zhao et al. (2007) |
| 24 | FliA | b1760 | microarray analysis | Zhao et al. (2007) |
| 45 | FliA | yjcZ | microarray-based genetic footprinting, microarray analysis | Girgis et al. (2007), Zhao et al. (2007) |
| 45 | FliA | yjdA | microarray-based genetic footprinting, microarray analysis | Girgis et al. (2007), Zhao et al. (2007) |
| 48 | GadE | yhiD | in RegulonDBv6.1 | Gama-Castro et al. (2008) |
| 48 | GadE | yhiU | in RegulonDBv6.1 | Gama-Castro et al. (2008) |
| 48 | GadE | slp | microarray analysis (induction by YdeO or GadE?) | Masuda & Church (2003) |
| 48 | GadE | yhiF | microarray analysis (induction by YdeO or GadE?), macroarray analysis | Masuda & Church (2003), Hommais et al. (2004) |
| 78 | LexA | dinP (dinB) | microarray analysis, ChIP-chip analysis | Courcelle et al. (2002), Wade et al. (2005) |

REFERENCES


Bradley, M. D., M. B.Beach, A. P. de Koning, T. S. Pratt, and R. Osuna, 2007, Microbiology **153**, 2922.
Courcelle, J., A. Khodursky, B. Peter, P. O. Brown, and P. C. Hanawalt, 2001, Genetics **158**, 41.
Faith, J. J., B. Hayete, J. T. Thaden, I. Mogno, J. Wierzbowski, G. Cottarel, S. Kasif, J. J. Collins, and T. S. Gardner, 2007, PLoS Biol **5**, e8.
Fernández De Henestrosa, A. R., T. Ogi, S. Aoyagi, D. Chafin, J. J. Hayes, H. Ohmori, and R. Woodgate, 2000, Mol Microbiol **35**(6), 1560.
Gama-Castro, S., V. Jiménez-Jacinto, M. Peralta-Gil, A. Santos-Zavaleta, M. I. Peñaloza-Spinola, B. Contreras-Moreira, J. Segura-Salazar, L. Muñiz-Rascado, I. Martínez-Flores, H. Salgado, et al., 2008, Nucleic Acids Res **36**(Database Issue), D120.
Girgis, H. S., Y. Liu, W. S. Ryu, and S. Tavazoie, 2007, PloS Genet **3**, e154 .
Grainger, D. C., H. Aiba, D. Hurd, D. F. Browning, and S. J. Busby, 2007, Nucleic Acids Res **35**, 269.
Hommais, F., E. Krin, J. Y. Coppée, C. Lacroix, E. Yeramian, A. Danchin, and P. Bertin, 2004, Microbiology **150**, 61.
Hung, S. P., P. Baldi, and G. W. Hatfield, 2002, J Biol Chem **43**, 40309.
Karp, P. D., I. M. Keseler, A. Shearer, M. Latendresse, M. Krummenacker, S. M. Paley, I. Paulsen, J. Collado-Vides, S. Gama-Castro, M. Peralta-Gil, et al., 2007, Nucleic Acids Res **35**, 7577.
Landgraf, J. R., J. A. Boxer, and J. M. Calvo, 1999, J Bacteriol **181**(20), 6547.
Masuda, N., and G. M. Church, 2003, Mol Microbiol **48**(3), 699.
Wade, J. T., N. B. Reppas, G. M. Church, and K. Struhl, 2005, Genes Dev **19**, 2619.
Weber, H., T. Polen, J. Heuveling, V. F. Wendisch, and R. Hengge, 2005, J Bacteriol **187**(5), 1591.
Zhao, K., M. Liu, and R. R. Burgess, 2007, Nucleic Acids Res **35**, 4441.


Table S1: New interactions predicted in the 30% precision LeMoNe network validated by literature search.
