# Supplementary material for: Comparative analysis of module-based versus direct methods for reverse-engineering transcriptional regulatory networks
Source: BMC Syst Biol. 2009 May 7;3:49. doi: 10.1186/1752-0509-3-49 (PMC2684101; doi:10.1186/1752-0509-3-49)
Supplement: Additional file 6 — Expression levels of chemotaxis and flagellar genes in E. coli. Supplementary Figure S5. [file 1752-0509-3-49-S6.pdf]

Supplementary Figure S5 – Expression levels of chemotaxis and flagellar genes in *E. coli*

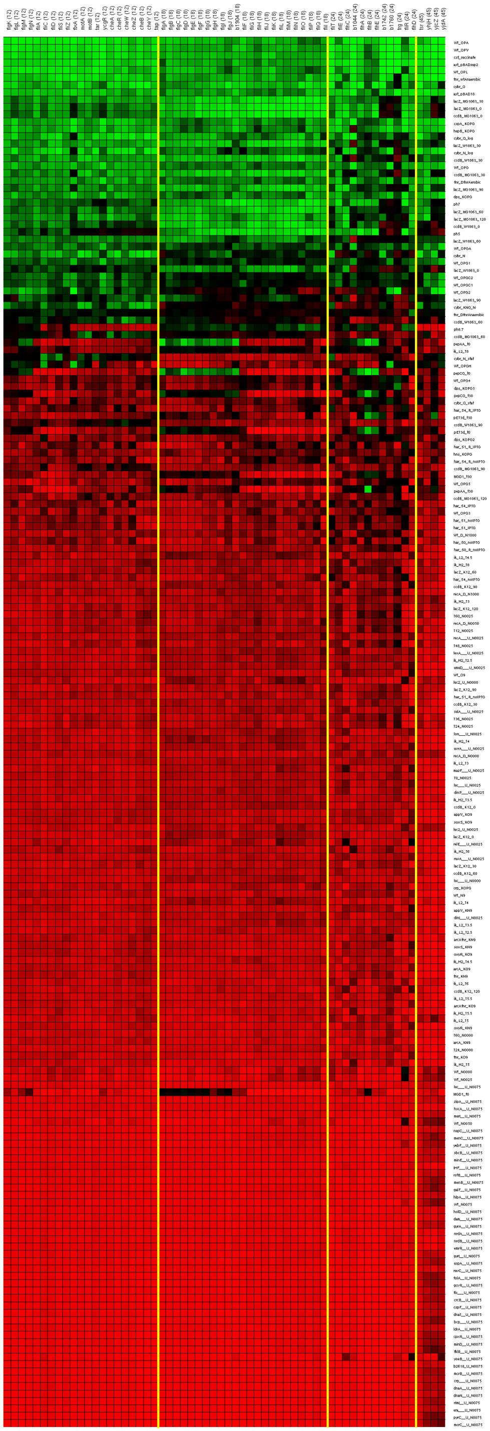

Figure S5: Expression levels of the genes in modules 12, 18, 24 and 45 for *E. coli*, with genes sorted in the same order as in Figure 5 (b). Conditions are sorted by the mean expression over all genes and yellow lines indicate module boundaries.
